# Hypereosinophilic Syndrome Following the BNT162b2 (BioNTech/Pfizer) Vaccine Successfully Treated with Mepolizumab: A Case Report and Review of the Literature

**DOI:** 10.3390/jcm12062376

**Published:** 2023-03-19

**Authors:** Ariela Hoxha, Tania Tomaselli, Giacomo Maria Minicucci, Jacopo Dall’Acqua, Davide Zardo, Paolo Simioni, Luigi Naldi

**Affiliations:** 1 General Internal Medicine, Hemorrhagic and Thrombosis Unit, Department of Medicine, University of Padua, 35128 Padua, Italy; 2 Internal Medicine Unit, Department of Medicine, San Bortolo Hospital, 36100 Vicenza, Italy; 3 Neurology Unit, San Bortolo Hospital, 36100 Vicenza, Italy; 4 Radiology Unit, San Bortolo Hospital, 36100 Vicenza, Italy; 5 Department of Pathology, San Bortolo Hospital, 36100 Vicenza, Italy; 6 Dermatology Unit, San Bortolo Hospital, 36100 Vicenza, Italy

**Keywords:** hypereosinophilic syndrome, vaccine, SARS-CoV-2, hypereosinophilia, mepolizumab

## Abstract

Although an increasing number of real-life data confirm large-scale clinical trial findings on the efficacy and safety of SARS-CoV-2 vaccines, rare but severe adverse reactions have begun to emerge. Here, we report a full-blown hypereosinophilic syndrome (HES) following a BNT162b2 (BioNTech/Pfizer) vaccine. A 48-year-old man developed, 5 days after the first shot of the SARS-CoV-2 vaccine, erythematous and painful nodular lesions in the lower and upper limbs accompanied by widespread itching, acrocyanosis with gangrenous lesions at the tips of the first and fourth fingers of the right hand, as well as paresthesia in the right hand and foot. Investigations revealed isolated eosinophilia, occlusion of the right ulnar artery, and electromyography alteration compatible with multifocal sensory neuropathy, as well as minimal accentuation of the interstitial texture with some ground glass appearance. Despite treatment with prednisone in combination with warfarin, he developed thrombosis of the left ulnar artery. Therefore, therapy with an IL-5 inhibitor and acetylsalicylic was successfully added. Given the time interval between the onset of clinical manifestations and the vaccine shot, we believe that the mRNA vaccine triggered the eosinophilic response. This case evidences a possible link between HES and the SARS-CoV-2 vaccination. Mepolizumab, an IL-5 inhibitor, might be considered in steroid refractory cases.

## 1. Introduction

Severe acute respiratory syndrome coronavirus 2 (SARS-CoV-2) infection continues scourging throughout the world, causing by January 2023 a total of 664 million confirmed coronavirus disease (COVID-19) infections with 6.7 million deaths [1]. Vaccination is the most compelling strategy to end this pandemic. Until now, a total of 13,131,550,798 vaccine doses have been administered across the continents [1]. An increasing amount of real-life data confirms findings of large-scale clinical trials on the efficacy and safety of those vaccines, even in specific clinical conditions [2,3,4,5]. On the other hand, as for other vaccinations, rare but severe adverse reactions have started to surface. Given the pandemic emergency, the approval of these vaccines was based on phase 2/3 trial data and a limited follow-up period. Therefore, the role of the scientific community is to observe and report any adverse effects. Here, we report a full-blown hypereosinophilic syndrome following a BNT162b2 (BioNTech/Pfizer, Mainz, Germany/New York, NY, USA) vaccine. The patient agreed to data publication and written consent was obtained. Moreover, a systematic review of the literature has been performed.

## 2. Case Description

**Figure 1:**
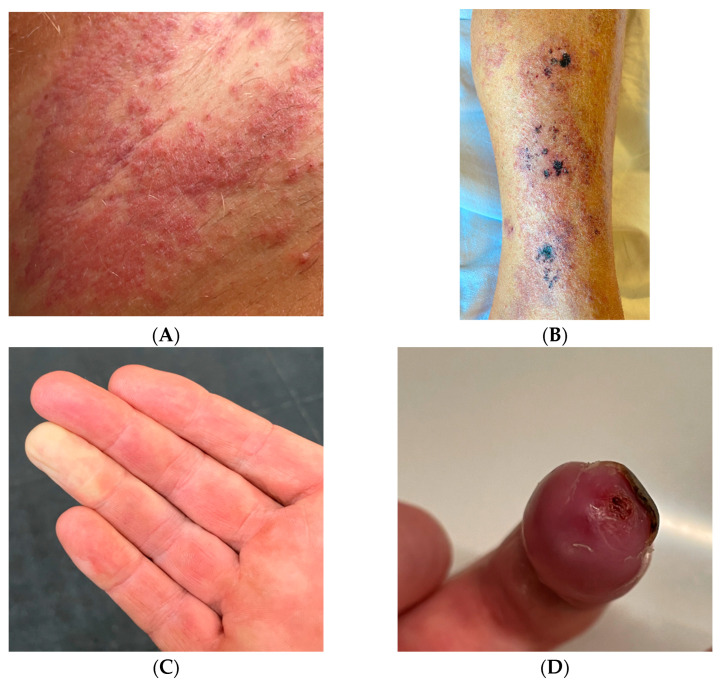
(**A**) Maculo-papular erythematous eruption on the trunk; (**B**) nodular purpuric lesions on the right leg; (**C**) Raynaud’s phenomenon; (**D**) acrocyanosis and gangrenous lesions at the tip of the fourth finger of the right hand.

**Figure 2:**
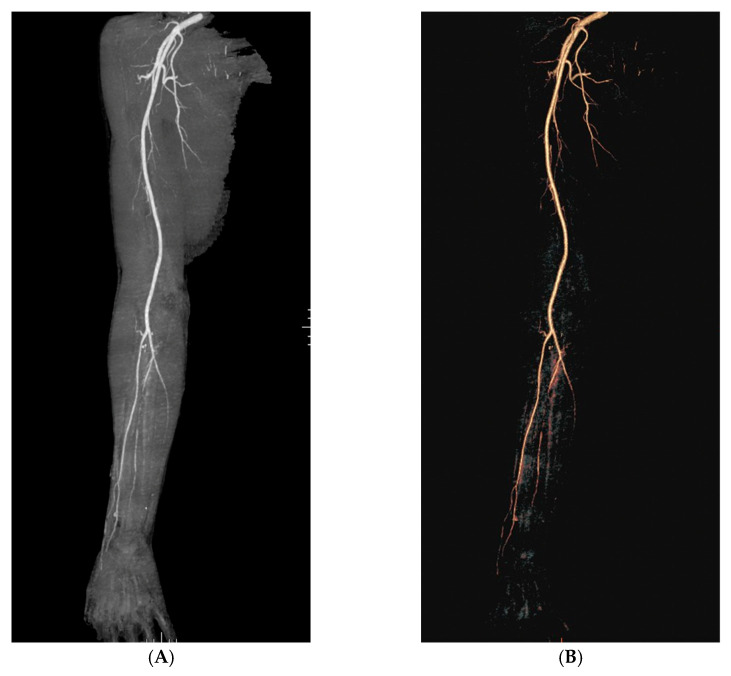
(**A**) Angio-TC 3D images MIP and (**B**) VRT demonstrating progressive thinning of the ulnar artery, occluded in its distal segment about at the half of the forearm.

**Figure 3:**
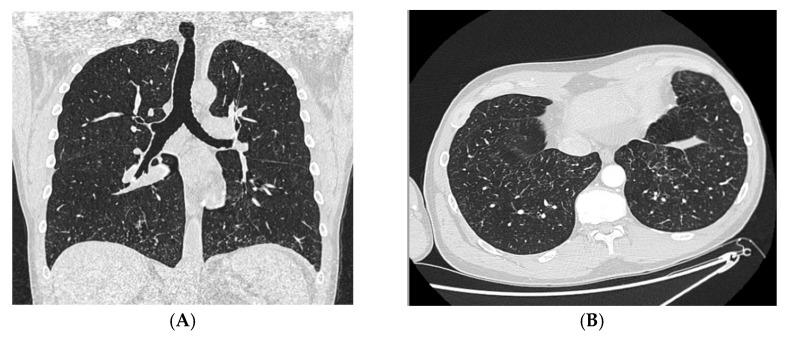
(**A**) HRCT coronal and (**B**) axial reconstructions demonstrating mild smooth thickening of the interlobular lung interstitium and the presence of many micronodules, especially in the basal sectors of the lower lobes.

**Figure 4:**
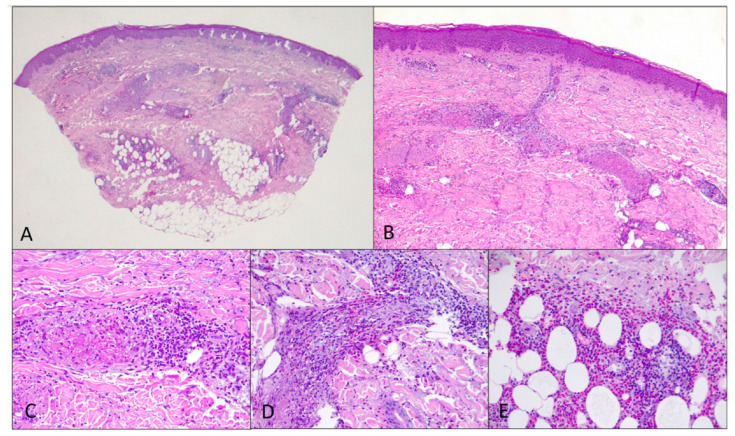
(**A**) Skin punch biopsy with unremarkable epidermis and underlying dermis showing prominent superficial and deep perivascular inflammation also involving subcutaneous adipose tissue (HE ×12.5); (**B**) venules of the superficial vascular plexus showing fibrinoid necrosis associated with numerous eosinophils, lymphocytes, and associated karyorrhectic debris (HE ×50); (**C**) and (**D**) close-up view of the vasculitis process (HE ×200); (**E**) close-up view of the heavy infiltrate of eosinophils in the subcutaneous adipose tissue (HE ×200).

A 48-year-old man with an unremarkable personal and family history, five days after the first shot of the BNT162b2 (BioNTech/Pfizer, Mainz, Germany/US) vaccine, experienced the onset of left inguinal adenopathy, and in the following days, erythematous dermatitis of the trunk (Figure 1A), treated unsuccessfully with topical steroids. Blood chemistry tests were unremarkable except for isolated eosinophilia (4.7 × 10^9^/L) and a slight increase in transaminases (GOT 57 U/L and GPT 132 U/L). An ultrasound examination of the groin showed increased reactive left inguinal lymph nodes (max diameter 1.6 cm). A course of azithromycin and acetaminophen was performed without symptomatic improvement. Lymph adenomegaly became bilateral, as shown by a new ultrasound of the groin (max diameter 2.4 cm) a week later. At the same time, erythematous and painful nodular lesions appeared in the lower and upper limbs accompanied by widespread itching (Figure 1B), as well as Raynaud’s phenomenon and acrocyanosis (Figure 1C) in addition to paresthesia in the right hand and foot. He underwent a hematological evaluation. A bone marrow biopsy and myelocentesis as well as immunophenotyping and karyotyping analyses were performed, all resulting normally. Moreover, the rearrangement of FIP1L1/PDGFRa was assessed and not found. Thus, a primary hematological disease was excluded. Due to the worsening of skin lesions and acrocyanosis with gangrenous lesions at the tips of the first and fourth fingers of the right hand (Figure 1D), the patient was admitted to the Internal Medicine ward of San Bortolo Hospital in Vicenza. A workup to evaluate the differential diagnosis was conducted. Thus, an angio-CT of the right upper limb (Figure 2) was performed showing the occlusion of the ulnar artery about 6.5 cm proximal to the radio-carpal rim. Furthermore, electromyography of the upper and lower limbs showed the absence of sensory nerve action potential (SNAP) of the right sural and median nerve as well as a reduced SNAP amplitude of the right and left ulnar nerves, compatible with multifocal sensory neuropathy (MSN). The Chest-HRCT (Figure 3) revealed the mild smooth thickening of the peripheral pulmonary interstitium with coexisting randomly distributed micronodules, especially at the lung bases, as well as some patchy “ground glass” opacities in the posterior basal sectors of the lower lobes, near the costophrenic sinuses. A pathological examination of a skin biopsy taken on the right leg showed a prominent superficial and deep perivascular inflammatory cell infiltrate including lymphocytes, numerous eosinophils, and sparse neutrophil polymorphs. Venules of the superficial vascular plexus also displayed fibrinoid necrosis associated with karyorrhectic debris (Figure 4A–D). An immunofluorescence test revealed the mild and focal perivascular granular deposition of IgA, C3c, and C1q. The ^18^FDG PET-CT was unremarkable, thus excluding malignancies. Laboratory examination showed increased immunoglobulin E (802 U/mL), thrombocytopenia (88 × 10^9^/L), and eosinophilia (8.4 × 10^9^/L), with unremarkable findings for C-reactive protein, search for fecal parasites, anti-myeloperoxidase and anti-proteinase 3 antineutrophil cytoplasmic antibody, antinuclear antibody, urinalyses, serum creatinine levels, radioallergosorbent test, serum tryptase, antiphospholipid antibodies, and congenital thrombophilia tests. All in all, a diagnosis of hypereosinophilic syndrome (HES) with vascular, neurological, pulmonary, and cutaneous involvement was made. Methylprednisolone 500 mg daily for 3 days, and then prednisone 1 mg/kg daily, was started in addition to anticoagulation with low molecular heparin bridging to warfarin. A few days after discharge, the patient attended the outpatient clinic with prominent acrocyanosis of the left hand. A Doppler ultrasonography of the left upper limb showed the occlusion of the third distal of the left ulnar artery confirmed by an angio-TC. Thus, therapy with an interleukin-5 (IL-5) inhibitor was discussed, and subcutaneous mepolizumab 300 mg every 4 weeks and acetylsalicylic acid 100 mg daily were added to the treatment regimen. The patient experienced a rapid improvement in clinical symptoms. The eosinophil count returned to normal, the skin lesions recovered, and he regained sensitivity in his right leg and hand. Electromyography, performed 3 months later than the previous one, showed a recordable SNAP of the right sural and median nerves, although still significantly reduced compared with the contralateral and normal SNAP amplitude of the right and left ulnar nerves. Therefore, after six months, mepolizumab was discontinued, and corticosteroid therapy was tapered progressively to prednisone 5 mg daily. Instead, warfarin and low-dose aspirin were continued. Almost a month later, the patient was presented to the outpatient clinic for erythematous and painful nodular lesions that appeared in the lower limbs accompanied by widespread itching. A new skin biopsy was performed. The pathologic examination showed a marked mixed inflammatory infiltrate comprising numerous eosinophilic granulocytes, mainly perivascular sites associated with aspects of vasculitis of small caliber vessels characterized by the fibrinoid necrosis of the wall in the dermo-hypodermic site. An increase in eosinophilic granulocytes was also observed in the interstitial area. Therefore, disease flares were confirmed. Then, therapy with mepolizumab 300 mg every 4 weeks was resumed, and the dosage of prednisone was increased to 25 mg daily with a prompt improvement of clinical symptoms. At the follow-up visit 12 months later, he was in complete remission. An upper limb Doppler ultrasonography demonstrated the recanalization of both the right and left ulnar arteries, so prednisone and warfarin were withdrawn. He continued therapy with mepolizumab 300 mg every 4 weeks and acetylsalicylic acid 100 mg daily. At the last follow-up in December 2022, he was doing well, and no signs or symptoms of HES were present.

## 3. Discussion

The HES are a heterogeneous group of disorders characterized by hypereosinophilia (>1.5 × 10^9^/L) and may be associated with life-threatening organ damage as a result of eosinophil infiltration [6] in various tissues and organs. In most cases, HES is associated with allergies, infections, medications, and autoimmune disorders. More rarely, it can be an expression of an underlying myeloid/lymphoid neoplasm. Thus, secondary causes must be excluded from the beginning as they represent the first step to making a precise diagnosis. Although any tissue or organ system can be affected by HES, eosinophilic infiltration is most observed in the cutaneous, pulmonary, gastrointestinal, cardiovascular, and neurologic organ systems [7]. The skin is the most frequently involved organ with cutaneous manifestations being the first presenting symptom of HES in more than 50% of cases [7]. Cardiovascular involvement affects approximately 40–60% of cases, followed by pulmonary, gastrointestinal, and neurological involvement [8]. Here, we have reported a case of HES developing 5 days after the first shot of the BNT162b2 mRNA (Pfizer/BioNTech, Mainz, Germany/US) vaccine, and presenting with vascular, pulmonary, neurologic, and cutaneous manifestations. Our patient’s first manifestation together with lymph adenomegaly was a maculopapular rash of the trunk, further involving the limbs as happens in most cases of HES. Additionally, the skin biopsy showed both superficial and deep perivascular inflammation also involving subcutaneous adipose tissue with the fibrinoid necrosis of superficial venules confirming a vasculitis process. Moreover, the clinical and neurophysiological data suggested a multifocal pure sensory involvement of the peripheral nervous system. As the motor part of mixed nerves (right median, right and left ulnar) was spared and because of the fast recovery, a possibly primary demyelinating/inflammatory process could be supposed. Indeed, peripheral neuropathies in the setting of HES have a variable presentation and may consist of isolated sensory or motor deficits or a mixture of both [8]. Thrombosis is another complication that can be observed in eosinophilic vasculitis. Our patients presented thrombosis of both ulnar arteries. Note that the thrombosis on the right ulnar artery happened while he was on treatment with steroids and warfarin. Finally, even though he did not complain of respiratory symptoms, the chest CT showed pulmonary involvement. In this scenario, our patient underwent a thorough differential diagnostic workup to evaluate the various etiologies of hypereosinophilia. We have excluded hematological disorders, infectious and autoimmune diseases, as well as allergic reactions to other drugs or foods. Thus, given the time interval between the onset of clinical manifestations and the vaccine shot, together with the absence of all other known causes of hypereosinophilia, we believe that the mRNA vaccine triggered the eosinophilic response, like some other viral infections or drugs [9]. Indeed, acute eosinophilic pneumonia was already described following influenza and pneumococcal vaccination [10,11]. Moreover, during the development of the respiratory syncytial virus vaccine some cases of fatal pulmonary eosinophilia after the vaccine challenge were reported [12]. The same phenomenon was observed in animal models of the anti-SARS-CoV1-1 vaccine [13]. Although mRNA vaccines, unlike recombinant vaccines, have no adjuvants, different stabilizers such as polyethylene glycol, polysorbates, and tromethamine/trometamol are reported to be potential stimulators of allergic hypersensitivity reactions [14]. Even though anaphylaxis is rare, other hypersensitivity reactions may be associated with COVID-19 vaccination.

However, a full-blown HES was never reported as far as we know. We performed a detailed search in the scientific databases Pubmed, Scopus, and EMBASE for original articles. The search strategy combined free text search, exploded medical subject headings (MESH/EMTREE) terms, and all synonyms of the following MESH terms to identify relevant published articles: “COVID-19”, “COVID-19 vaccines”, and “mRNA vaccines” in combination with “hypereosinophilic syndrome”. We identified a total of seven cases of hypereosinophilic conditions following the anti-SARS-CoV-2 vaccine and summarized the patients’ age, gender, clinical presentation, treatment, and outcome (Table 1) [15,16,17,18,19,20].

There were five cases of pulmonary eosinophilia and two cases of HES [16,18,19,20]. One of the HES cases presented with eosinophilic myocarditis and periorbital and pitting edema [17]. The patient, a 33-year-old man, after 8 days post-vaccination with the first dose of COVAXIN (Bharat Biotech, Telangana, India) developed swelling initially under both eyes and then on his hands, and on day 25 of vaccination, claimed shortness of breath. Cardiac magnetic resonance imaging showed hyperintense areas and edema in the left ventricular myocardium on the T2 image suggestive of myocarditis [17]. In the other case, a 73-year-old woman presented with pulmonary eosinophilia and cutaneous involvement on day 4 of the second dose of the CoronaVac (Sinovac, Beijing, China) vaccination [15]. She had started coughing after the first dose of the vaccine as an isolated symptom and then manifested with maculopapular rash and dyspnea after the second dose [15]. Lung biopsies showed eosinophilic infiltration [15]. However, unlike our case, the skin biopsy displayed edema of the superficial dermis and a dense infiltrate of lymphocytes, but not eosinophilic infiltrate. Overall, the clinical manifestations developed from 5 h to 7 weeks after COVID-19 vaccination. The mRNA vaccine as well as those with inactivated SARS-CoV-2 virus were involved in hypereosinophilic conditions.

All cases developed clinical manifestations after the first shot of the COVID-19 vaccination, except one case. Peripheral blood eosinophils were lower than the threshold (1.5 × 10^9^/L) in two cases; however, both showed higher levels of eosinophils in bronco-alveolar lavage (BAL), respectively, 50% and 11%. Five of the seven cases demonstrated eosinophilic infiltration at BAL or at cutaneous and/or lung biopsy. All the cases, except one, which improved spontaneously, were treated successfully with intravenous corticosteroids. Despite corticosteroid therapy, our patient presented a worsening clinical picture. Even though the mechanism of eosinophil overproduction and tissue accumulation was not fully understood yet, IL-5 has been recognized as the key inflammatory cytokine involved in the priming and survival of eosinophils and in their proliferation and maturation [21]. For this reason, we started treatment with mepolizumab, an IL-5 inhibitor, observing a progressive and accelerated clinical improvement and decrease in eosinophil count.

## 4. Conclusions

The purpose of our case study is to highlight the evidence linking HES with SARS-CoV-2 vaccination, pointing to the need for a timely diagnosis and early appropriate treatment. As to other vaccines/medications, immune reactions are possible, so it is important to notice these reactions and to closely follow up on the subjects who may develop such reactions. Finally, our work conveys the message of the efficacy of mepolizumab, an IL-5 inhibitor in steroid-refractory HES.

## Figures and Tables

**Table 1:** Summary of hypereosinophilic syndrome after COVID-19 vaccine in the literature.

Author, Year	Sex	Age (Years)	COVID-19 Vaccine	Time from Vaccine	Clinical Condition	Blood Eosinophils	Biopsy	Treatment	Outcome
Ozturk et al., 2023 [15]	F	73	Sinovac/CoronaVac	4 days	HES	2300 μ/L	BAL: 36% eosinophils TBNA: eosinophile infiltration	MPN 1 mg/kg/day for 7 days and then 40 mg/daily tapered and discontinued over 3 months	Recovered with steroids
May et al., 2022 [16]	F	55	ChAdOx1 nCov-19	7 weeks	Eosinophilic pneumoniae	900 μ/L	BAL: 50% eosinophils	MPN 500 mg then PDN 30 mg per day, tapered and discontinued over 12 weeks	Recovered with steroids
Tiwari et al., 2022 [17]	M	33	COVAXIN	8 days	HES	2767 μ/L	NA	Dexa 6 mg twice daily for five days	Recovered with steroids
Costa e Silva et al., 2022 [18]	F	38	NR	2 weeks	Eosinophilic pneumoniae	3250 μ/L	BAL: 13% eosinophils	Deflazacort 60 mg/die	Recovered with steroids
	F	47	NR	4 weeks	Eosinophilic pneumoniae	800 μ/L	BAL: 11% eosinophils	PDN 40 mg/day for 14 days	Recovered with steroids
Piqueras et al., 2022 [19]	M	37	Pfizer-BioNTech	8–10 h	Eosinophilic pneumoniae	4130 μ/L	BAL: 60.3% eosinophils	None	Spontaneous clinical improvement
Miqdadi et al., 2021 [20]	M	66	ChAdOx1 nCoV-19	5 h	Eosinophilic pneumoniae	3960 μ/L	NA	MPN 240 mg/daily for 5 days and then tapered and discontinued over 3 months	Recovered with steroids

HES: hypereosinophilic syndrome; BAL: bronchoalveolar lavage; TBNA: transbronchial needle aspiration; NR: not reported; NA: not available, MPN: methylprednisolone; PDN: prednisolone; Dexa: dexamethasone.

## Data Availability

The data presented in this study are available in this article.
